# pFIB scores for paediatric MASLD fibrosis are associated with risk of youth-onset type 2 diabetes in children with obesity

**DOI:** 10.1007/s00125-026-06804-4

**Published:** 2026-07-22

**Authors:** Sander Lefere, Resthie R. Putri, Pernilla Danielsson, Thomas Casswall, Claude Marcus, Christian A. Hudert, Anja Geerts, Ruth De Bruyne, Emilia Hagman

**Affiliations:** 1 Hepatology Research Unit, Department of Internal Medicine and Pediatrics, Ghent University, Ghent, Belgium; 2 Liver Research Center Ghent, Ghent University Hospital, Ghent, Belgium; 3 Department of Clinical Science, Intervention and Technology, Karolinska Institutet, Stockholm, Sweden; 4 Department of Medical Epidemiology and Biostatistics, Karolinska Institutet, Stockholm, Sweden; 5 Department of Pediatric Gastroenterology, Nephrology and Metabolic Diseases, Charité - Universitätsmedizin Berlin, Berlin, Germany; 6 Department of Pediatric Gastroenterology, Hepatology and Nutrition, Ghent University Hospital, Ghent, Belgium

**Keywords:** Biomarker, Children, Paediatric obesity, Prognostic, Risk score

## Abstract

**Aims/hypothesis:**

Youth-onset type 2 diabetes is an aggressive phenotype of metabolic disease, yet tools to identify children with obesity at highest short-term risk are limited. We evaluated whether the paediatric liver fibrosis risk scores (pFIB) pFIB-c and pFIB-6, originally developed for metabolic dysfunction-associated steatotic liver disease (MASLD) fibrosis risk stratification, identify individuals at increased risk of incident type 2 diabetes.

**Methods:**

A cohort study was conducted using the Swedish Childhood Obesity Treatment Register (BORIS, 2005–2020) linked with national registers, from which type 2 diabetes was ascertained. We included 4856 children with obesity (aged 12.0 years at baseline) and 23,758 general population comparators. The pFIB-c and pFIB-6 scores were calculated as published. Time-to-event analysis yielding HR was performed using Cox regression and flexible parametric survival models.

**Results:**

Among children with obesity, 12.4% had a pFIB-6 ≥4. A high pFIB-6 score increased the risk for type 2 diabetes at 9–19 years of age (adjusted HR 3.05 [95% CI 2.17, 4.28]). The incidence rate (95% CI) per 10,000 person-years was 1.02 (0.61, 1.72) in the general population comparators, 45.5 (37.8, 54.7) in individuals with obesity and a pFIB-6 <4, and 161.7 (122.2, 213.9) when the pFIB-6 was ≥4. In contrast, neither score, measured at paediatric age, predicted type 2 diabetes risk at 20–25 years of age. Among individual score components, elevated alanine aminotransferase (ALT) and HOMA-IR were most strongly associated with type 2 diabetes risk. In an independent cohort, higher pFIB-6 scores were associated with greater adipose tissue insulin resistance.

**Conclusions/Interpretation:**

A liver-derived composite risk score identifies a subgroup of adolescents with obesity at markedly increased short-term risk of type 2 diabetes. These findings suggest that integrating hepatic and metabolic variables may improve early risk stratification in paediatric obesity and help identify individuals who may benefit from intensified monitoring during adolescence.

**Graphical Abstract:**

**Figure:**
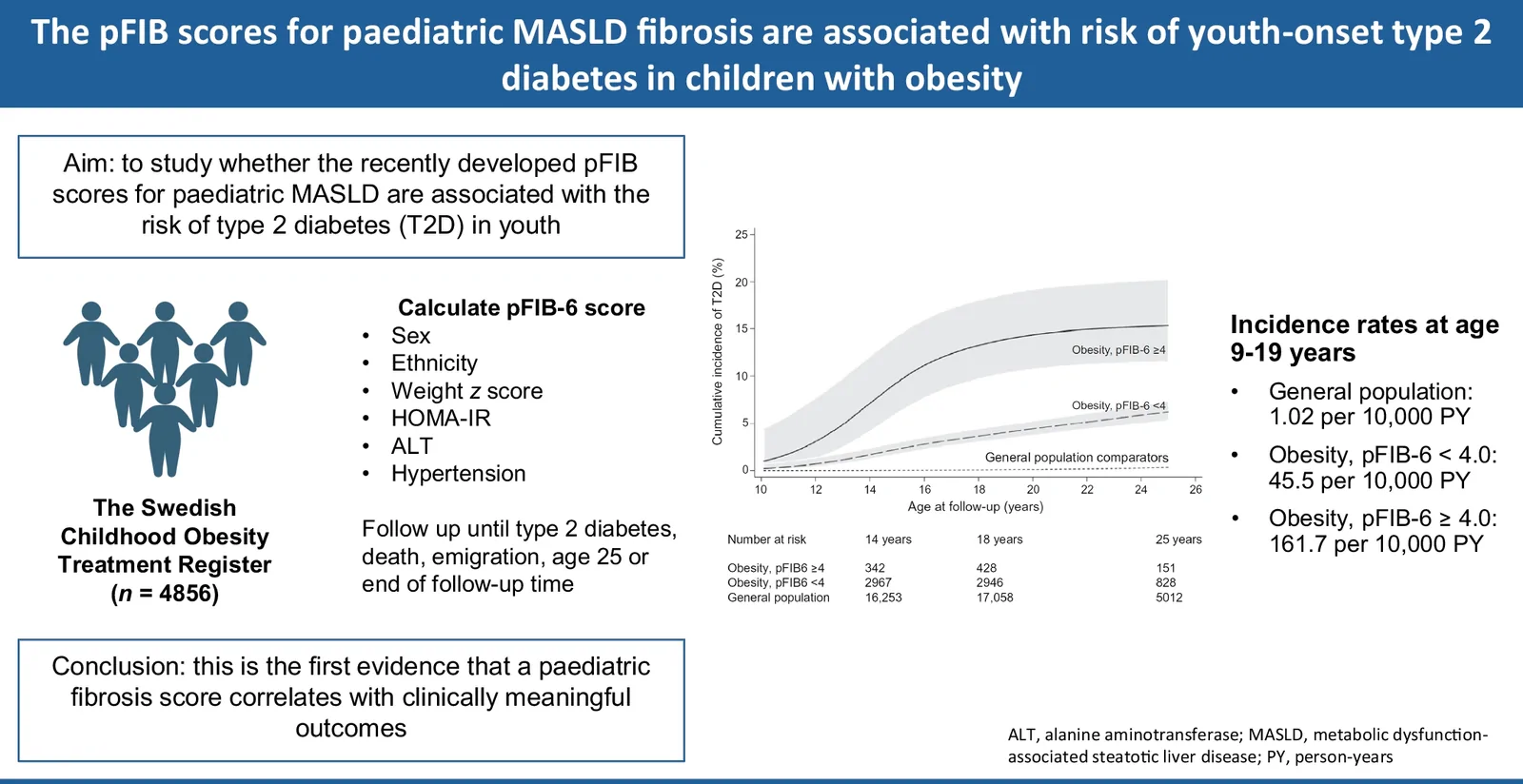

**Supplementary Information:**

The online version of this article (https://doi.org/10.1007/s00125-026-06804-4) contains peer-reviewed but unedited supplementary material.

**Figure:**
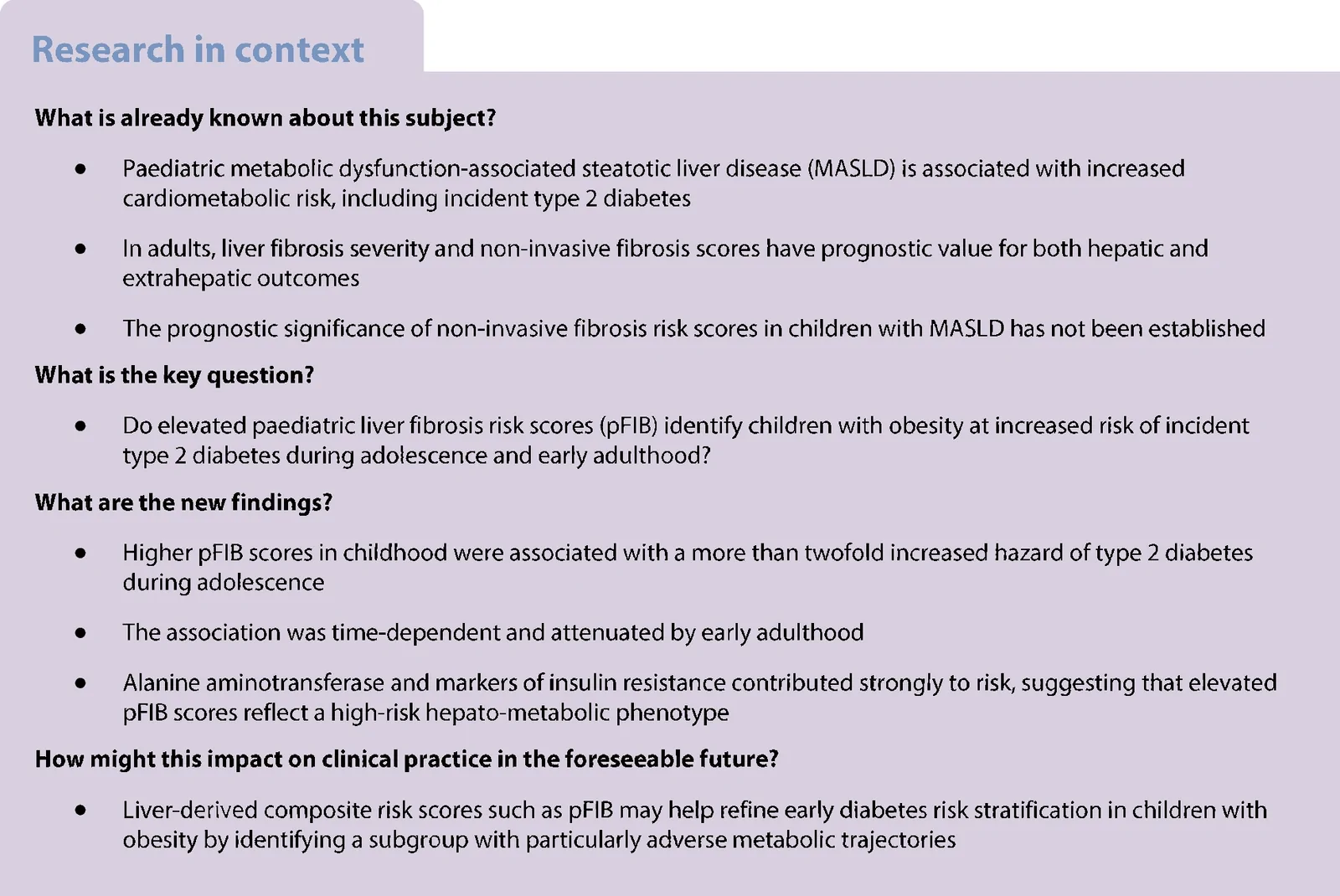

## Introduction

The global rise in childhood obesity has been accompanied by a marked increase in metabolic dysfunction-associated steatotic liver disease (MASLD, previously termed NAFLD), which now affects an estimated 7–14% of the general paediatric population and substantially more children with obesity [1]. MASLD reflects systemic metabolic dysregulation and insulin resistance, and is increasingly recognised as a hepatic manifestation of cardiometabolic disease rather than an isolated liver condition.

In adults, the presence and severity of liver fibrosis are key determinants of long-term outcomes, including major adverse liver outcomes (MALO), cardiovascular disease and overall mortality [2, 3]. Emerging data suggest that paediatric MASLD is likewise associated with increased risks of premature mortality and major adverse cardiovascular events in young adulthood [4, 5], particularly in individuals with steatohepatitis and/or fibrosis [4, 6]. However, the natural history of fibrosis in children remains incompletely defined and liver-related events are rare during adolescence [7].

In parallel, accumulating evidence indicates that MASLD in childhood confers a substantially increased risk of incident type 2 diabetes, especially in those with more advanced histological severity [8, 9]. This is of particular concern given the aggressive course of youth-onset type 2 diabetes, characterised by rapid beta cell decline and early complication development [10, 11]. The bidirectional relationship between MASLD and type 2 diabetes, with diabetes also accelerating liver disease progression [12], as well as the lack of longitudinal clinical prediction models for estimating future type 2 diabetes risk in initially non‑diabetic youth [13], further underscore the need to identify children at highest metabolic risk.

Non-invasive tools to assess fibrosis are central to risk stratification in adult MASLD, where scores such as FIB-4 and vibration-controlled transient elastography provide prognostic information beyond liver-related outcomes [14, 15]. In contrast, existing non-invasive fibrosis scores perform suboptimally in paediatric populations [16]. To address this gap, we recently developed and validated a composite fibrosis risk score for paediatric MASLD (continuous pFIB-c and the simplified pFIB-6), designed to identify children with significant liver fibrosis [17]. The relatively low incidence of MALO in paediatric MASLD [18], combined with the requirement for extended follow-up, limits the ability to demonstrate comparable prognostic utility of paediatric risk scores.

Whether such a liver-derived risk score also identifies children at increased risk of clinically meaningful metabolic outcomes has not been established. We therefore hypothesised that elevated pFIB scores are associated with a higher risk of incident type 2 diabetes during adolescence and early adulthood.

## Methods

### Study design

Data from a cohort study of children undergoing obesity treatment, who were enrolled in the nationwide Swedish Childhood Obesity Treatment Register (BORIS, https://www.e-boris.se/in-english/, accessed 1 February 2026) from 2005 to 2020, were analysed. This register covers paediatric obesity treatment at all healthcare levels across Sweden.

The study population consisted of children with obesity according to International Obesity Task Force criteria [19], aged 9–18 years at enrolment, free from type 2 diabetes at baseline and with available data on all variables necessary to calculate the pFIB scores [17], as detailed below. Children were excluded if, according to the ICD-10 codes, there was evidence of other causes of steatotic liver disease or increased alanine aminotransferase (ALT), such as viral or auto-immune hepatitis, α_1_-antitrypsin deficiency, drug-induced liver injury, coeliac disease and inflammatory bowel disease, secondary causes of hypertension, or whether children were diagnosed with type 1 diabetes, MODY or genetic syndromes related to obesity (e.g. Prader–Willi syndrome, Bardet–Biedl syndrome or Down syndrome). The full list of exclusion criteria and corresponding ICD-10 codes is presented in electronic supplementary material (ESM) Table 1. General population comparators were matched to the obesity cohort in a 1:5 ratio, based on sex, birth year and residential area. The cohort was followed from age 9 years or from the age at obesity treatment initiation until one of the following events occurred: type 2 diabetes; emigration; death; age 25 years; or the end of follow-up (July 2023).

The research was conducted in accordance with both the Declarations of Helsinki and Istanbul. Ethical approval was obtained from the Regional Ethical Review Board in Stockholm (Dnr 2016/922–31/1; 2020–02707).

### Data sources

The BORIS register records clinical and laboratory visits in paediatric obesity centres across Sweden of patients both with and without obesity comorbidities [20]. At baseline, patients received lifestyle intervention for obesity. At that time, incretin-based obesity medications were not approved for adolescents in Sweden and, thus, no patient received pharmacotherapy for obesity. BORIS was linked with several Swedish national registers maintained by the Swedish National Board of Health and Welfare via the Swedish unique personal identity number. First, the National Patient Register, which covers inpatient and specialised outpatient care, was utilised to obtain medical diagnoses of type 2 diabetes, secondary causes of steatosis, and genetic syndromes. In general, the positive predictive value of the majority of diagnoses in this register varies from 85% to 95% [21]. Second, the Prescribed Drug Register was used to identify the prescription of glucose-lowering medications in any level of care. Date of death or migration data were obtained from the Cause of Death Register and Total Population Register, respectively; the latter was also used to collect general population comparators and demographic variables.

### Exposure, outcome and confounding variables

The main exposure variables were the pFIB scores, namely the pFIB-c score and the pFIB-6 score, as described previously [17]. The pFIB-c score is a probabilistic score ranging from 0 to 1, reflecting the likelihood of significant liver fibrosis. It is calculated as e^x^/(1 + e^x^), with x=−5.155 + 1.194 × sex (boy=1) + 1.636 × hypertension (yes=1) + 0.019 × ALT + 0.633 *×* weight *z* score + 0.079 *×* HOMA-IR −2.453 *×* black ethnicity (yes=1). An online calculator is available on MDCalc (https://www.mdcalc.com/calc/10615/paediatric-fibrosis-score-continuous-pFIB-c). To facilitate adoption in a clinical setting, a simplified and categorised pFIB-6 score was derived [17]. The pFIB-6 ranges between −1 and +6, with the following components: sex (boy=1); hypertension (present=2); black ethnicity (yes=−1); ALT (> upper limit of normal [ULN]=0.5 points; >2 *×* ULN=1 point); HOMA-IR (≥3=0.5 points; ≥4.5=1 point); and weight *z* score (≥2=0.5 points; ≥3=1 point). Children with missing data precluding calculation of the scores were excluded.

Variables constituting the pFIB scores were collected from the above-described registers. A child’s sex was categorised as male or female based on legal sex in the Total Population Register. For the clinical components of the score, the first measurements recorded in BORIS were used. Weight status was expressed as weight *z* score and BMI *z* score based on the British 1990 growth reference [22]. The ALT ULN was defined as 22U/l in girls and 26U/l in boys [23]. HOMA-IR was calculated as the product of fasting insulin (pmol/l) × fasting glucose (mmol/l)/135. For hypertension, cut-off values proposed by Lurbe et al were used [24]. These are based on reference values in lean children. Hypertension was defined by a BP >95th percentile corresponding to the child’s sex, age and height or use of BP-lowering medication. Parental country of birth as recorded in the Total Population Register was used as a proxy for ethnicity. Black ethnicity was defined as having at least one parent born on the African continent.

As the main outcome variable, type 2 diabetes was identified using the Swedish National Patient Register (ICD-10 code E11 [type 2 diabetes] or O24.1 [preexisting type 2 diabetes mellitus, in pregnancy, childbirth and the puerperium]) and/or glucose-lowering medications according to the Swedish Prescribed Drug Register using an algorithm described in ESM Table 2 and previously detailed in Putri et al [8].

Parental type 2 diabetes, an important confounding factor and proxy for heredity, was defined as at least one parent who had developed type 2 diabetes (from the National Patient Register and/or Prescribed Drug Register) before the age of 50 years, as described previously [8].

### Validation cohort for adipose tissue insulin resistance

Children and adolescents with severe obesity (*n*=200 included in this analysis) were recruited at the Zeepreventorium (De Haan, Belgium) between July 2020 and December 2022. Eligibility criteria included BMI exceeding the age- and sex-specific cut-off corresponding to an adult BMI of 30 kg/m^2^, the presence of at least two obesity-associated comorbidities, and previous failure or unfeasibility of ambulatory multidisciplinary management. Participants with evidence of syndromic obesity or other known causes of liver disease, including viral hepatitis and auto-immune liver disease, were excluded. The study was conducted in accordance with both the Declarations of Helsinki and Istanbul and was approved by the institutional ethics committee of Ghent University Hospital (BS-05660) [25]. All participants signed informed consent or gave assent; parents signed an informed consent.

Fasting blood samples were obtained after an overnight fast. Adipose tissue insulin resistance (Adipo-IR) was quantified by the Adipo-IR index, calculated as fasting insulin (pmol/l) × NEFA (mmol/l) × 6. Liver steatosis and fibrosis were evaluated using ultrasound and transient elastography with controlled attenuation parameter, performed by a single experienced operator (SL) using the FibroScan Mini+ 430 (Echosens, Paris, France) with M- or XL-probe according to body size. The pFIB-6 score was calculated as described. Additional methodological details are described in [25].

### Statistical analysis

Patient characteristics are reported as median (quartile 1 [Q1] to quartile 3 [Q3]) for continuous variables, and frequency and proportion for categorical variables. Individuals with obesity with and without elevated pFIB-6 scores were compared using the χ^2^ and independent *t* tests for categorical and continuous data, respectively.

The incidence rates per 10,000 person-years were calculated for the general population and individuals with obesity with or without an elevated pFIB score. To investigate the relationship between pFIB scores and risk of type 2 diabetes, time-to-event analysis yielding HR was performed using Cox regression and flexible parametric survival models. Both an unadjusted model and a model adjusted for the covariates age at baseline and parental type 2 diabetes were calculated.

First, the incremental risk associated with discrete pFIB test results was investigated, compared with the reference category of low pFIB scores (i.e. pFIB-6 ≤1 and pFIB-c <0.1). In a second step, we assigned dichotomous cut-offs of 0.4 and 4.0 to the pFIB-c and pFIB-6, respectively, as these corresponded to a high positive predictive value of MASLD fibrosis in a secondary analysis of the data from the original study [17].

Since a visual inspection of the Kaplan–Meier graph and the Schoenfeld residuals indicated that the HRs for type 2 diabetes were not proportional over time, split HRs during age 9–19 years and 20–25 years were also estimated.

We performed two sensitivity analyses. First, HRs for the individual pFIB score components and risk of type 2 diabetes were calculated. Second, the association between the pFIB scores and incidence of type 2 diabetes was stratified by sex.

Statistical analyses were performed using Stata version 18 and Graphpad Prism 10.5 (Graphpad Software, La Jolla, USA).

## Results

### Study population

At baseline, we included 4856 individuals in the obesity cohort for whom all variables were available to calculate the pFIB scores. They were matched 1:5 with 23,758 general population comparators. In the obesity cohort, a pFIB-6 score above the cut-off of ≥4 was present in 602 (12.4%) individuals. As expected from the score characteristics, individuals with a high pFIB-6 score had higher weight *z* score, BP, HOMA-IR and ALT levels than those with a low pFIB-6 score (Table 1). Moreover, parental type 2 diabetes was more prevalent in the high-score group.

**Table 1:** Baseline characteristics of the obesity cohort, stratified by pFIB-6 score, and of the general population comparators

Variable	Obesity cohort (*n*=4856)	pFIB-6 <4 (*n*=4254)	pFIB-6 ≥4 (*n*=602)	*p* value	General population comparators (*n*=23,758)
Sex, female	2069 (42.6)	1910 (44.9)	159 (26.4)	<0.001	10,115 (42.6)
Age at baseline, years	12.0 (10.3–14.1)	11.8 (10.3–14.0)	13.0 (10.9–14.9)	<0.001	12.0 (10.4–14.2)
Age at end of follow-up, years	20.3 (17.3–24.0)	20.3 (17.2–24.0)	20.6 (17.5–25.0)		20.5 (17.5–24.2)
Length of follow-up, years	7.8 (5.2–10.4)	7.9 (5.3–10.5)	7.5 (4.6–10.2)		8.2 (5.4–11.8)
Parental type 2 diabetes	703 (14.5)	587 (13.8)	116 (19.3)	<0.001	1329 (5.6)
Anthropometry					
Weight, *z* score	2.83 (2.47–3.30)	2.79 (2.44–3.21)	3.27 (2.91–3.74)	<0.001	
BMI, *z* score	2.73 (2.51–3.00)	2.69 (2.48–2.95)	3.02 (2.75–3.28)	<0.001	
Class 1 obesity	3170 (65.3)	2935 (69.0)	235 (39.0)		
Class 2 or 3 obesity	1686 (34.7)	1319 (31.0)	367 (61.0)	<0.001	
BP					
Systolic BP, mmHg	115 (108–122)	113 (107–120)	130 (124–137)	<0.001	
Diastolic BP, mmHg	69 (62–75)	68 (61−73)	76 (69–83)	<0.001	
Hypertension	811 (16.7)	296 (7.0)	515 (85.6)	<0.001	
Biochemistry					
Fasting glucose, mmol/l	5.22 (4.88-5.49)	5.22 (4.88-5.49)	5.27 (5.0-5.61)	<0.001	
Impaired fasting glucose	232 (4.8)	191 (4.5)	41 (6.8)	0.012	
HOMA-IR	3.8 (1.7–6.0)	3.5 (1.5–5.6)	5.9 (4.0–8.6)	<0.001	
ALT, U/l	24 (19–35)	23 (18–32)	35 (25–58)	<0.001	
ALT >2× ULN	519 (10.7)	326 (7.7)	193 (32.1)	<0.001	

Continuous variables are presented as median (Q1–Q3) and categorical variables as *n* (%)

*p* values reflect the comparison between individuals with a pFIB-6 <4 and those with a pFIB-6 ≥4

### Association between pFIB scores and risk for type 2 diabetes

A total of 201 (4.1%) individuals in the obesity cohort developed type 2 diabetes during a median follow-up time of 8.1 years, compared with 34 (0.14%) individuals in the general population comparators in a similar timeframe (median follow-up time 8.2 years). Of the 201 individuals in the obesity cohort who developed type 2 diabetes, 52 individuals had pFIB-6 ≥4 and 67 a pFIB-c >0.4.

Compared with individuals with a pFIB-6 score ≤1, the risk of diabetes did not increase in the fully adjusted model for test scores of 1.5–2 and 2.5–3, while there was a trend in individuals with a score of 3.5–4 (HR 1.54 [95% CI 0.91, 2.61]; *p*=0.108) (ESM Table 3). Risk was significantly elevated, however, in individuals with pFIB-6 ≥4.5 (HR 3.28 [95% CI 1.98, 5.44]; *p*<0.001). A similar pattern was observed when analysing the pFIB-c score in bins of 0.10 (ESM Table 3).

**Fig. 1:**
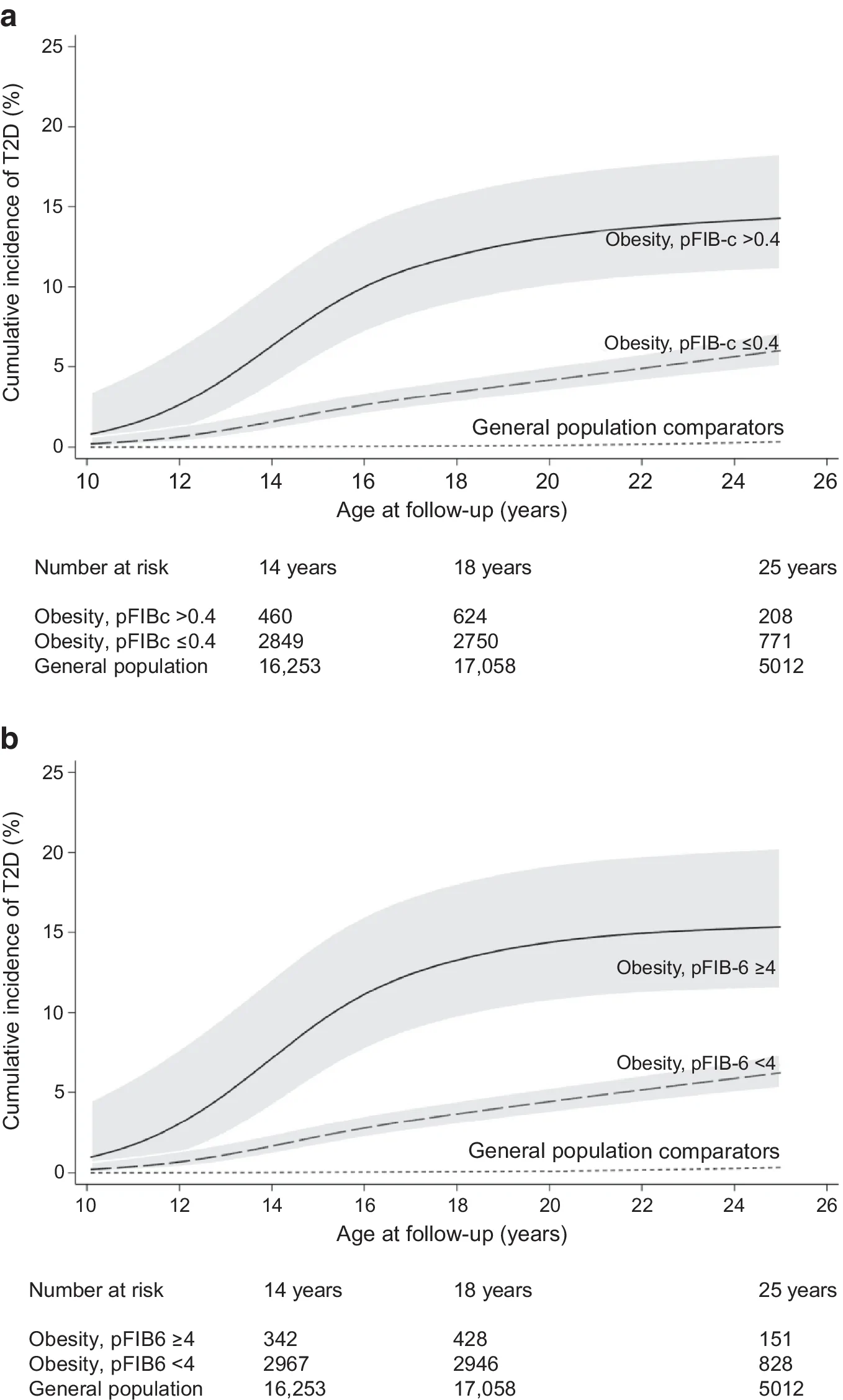
Cumulative incidence of type 2 diabetes by pFIB-c score (**a**) or pFIB-6 score (**b**). T2D, type 2 diabetes. The grey shading shows 95% CI

We next categorised individuals with obesity in low-pFIB and high-pFIB groups. The type 2 diabetes incidence rate was 1.8 (95% CI 1.3, 2.5) per 10,000 person-years in the general population comparators, 43.4 (95% CI 36.9, 50.9) in individuals with obesity and a pFIB-6 <4, and 114.4 (95% CI 87.2, 150.2) in those with obesity and a pFIB-6 ≥4 (cumulative incidence of type 2 diabetes is illustrated in Fig. 1).

These incidence rate data translated into a significantly increased risk of diabetes in individuals with a pFIB-6 ≥4, with an adjusted HR of 2.42 (95% CI 1.76, 3.32; *p*<0.001) (Table 2). The pFIB-c at a cut-off of 0.4 indicated a comparably increased risk, as the adjusted HR equalled 2.32 (95% CI 1.72, 3.13; *p*<0.001).

**Table 2:** Association between the pFIB score and incidence of type 2 diabetes

Score	*n* T2D/*n* total	Unadjusted HR (95% CI)	*p* value	Adjusted HR^a^ (95% CI)	*p* value
Age 9–25 years					
pFIB-6 score <4	149/4254	Ref		Ref	
pFIB-6 score ≥4	52/602	2.68 (1.95, 3.68)	<0.001	2.42 (1.76, 3.32)	<0.001
pFIB-c score ≤0.4	134/4011	Ref		Ref	
pFIB-c score >0.4	67/845	2.61 (1.94, 3.50)	<0.001	2.32 (1.72, 3.13)	<0.001
Age 9–19 years					
pFIB-6 score <4		Ref		Ref	Ref
pFIB-6 score ≥4		3.54 (2.53, 4.95)	<0.001	3.05 (2.17, 4.28)	<0.001
pFIB-c score ≤0.4		Ref		Ref	
pFIB-c score >0.4		3.58 (2.61, 4.92)	<0.001	3.03 (2.19, 4.19)	<0.001
Age 20–25 years					
pFIB-6 score <4		Ref		Ref	
pFIB-6 score ≥4		0.45 (0.16, 1.27)	0.133	0.53 (0.17, 1.72)	0.293
pFIB-c score ≤0.4		Ref		Ref	
pFIB-c score >0.4		0.51 (0.16, 1.66)	0.266	0.48 (0.17, 1.35)	0.162

HRs were estimated using Cox regression

^a^Adjusted for age at baseline and parental type 2 diabetes

T2D, type 2 diabetes

### Risk of type 2 diabetes over time

Table 2 and Fig. 1 show that the association between pFIB-c score and type 2 diabetes was stronger at ages 9–19 years than in later years. A high pFIB-c score increased the risk for type 2 diabetes at 9–19 years of age (HR 3.03 [95% CI 2.19, 4.19]; *p*<0.001). The type 2 diabetes incidence rate was 1.02 (95% CI 0.61, 1.72) per 10,000 person-years in the general population comparators; in individuals with obesity the incidence rate per 10,000 person-years was 45.5 (95% CI 37.8, 54.7) in individuals with a pFIB-6 <4 and 161.7 (95% CI 122.2, 213.9) in those with a pFIB-6 ≥4.

In contrast, neither pFIB-c score nor pFIB-6 score, measured at paediatric age, was associated with type 2 diabetes risk at 20–25 years of age (Table 2).

Consistent with the relative risk gradient across pFIB categories, the cumulative incidence of type 2 diabetes over time was nearly identical for individuals with low (<0.1) and intermediate (0.1–0.4) baseline pFIB-c scores, and these trajectories lay between those of the general population comparators and individuals with pFIB‑c ≥0.4 (ESM Fig. 1).

### Sensitivity analyses

We next calculated adjusted HRs for the pFIB-6 components to gauge the relative contribution made by the various factors to the observed association between the overall score and type 2 diabetes (Table 3). Hypertension, an elevated ALT (both >1× ULN and >2× ULN) and HOMA-IR ≥4.5 were associated with a greater likelihood of incident type 2 diabetes. In contrast, the inclusion of male sex in the scores decreased their performance, as girls were at higher risk of type 2 diabetes.

**Table 3:** Association between the individual components of the pFIB-6 scores and incidence of type 2 diabetes

pFIB-6 score component	Unadjusted HR (95% CI)	Adjusted HR^a^ (95% CI)
Sex, male	0.60 (0.45, 0.79)	0.58 (0.44, 0.77)
Hypertension	1.73 (1.27, 2.38)	1.43 (1.04, 1.97)
Black ethnicity	1.12 (0.65, 1.93)	1.20 (0.70, 2.08)
ALT		
ULN < ALT ≤2× ULN	1.69 (1.23, 2.34)	1.57 (1.13, 2.17)
ALT >2× ULN	3.94 (2.73, 5.70)	3.42 (2.34, 5.00)
HOMA-IR		
3 ≤ HOMA-IR <4.5	1.09 (0.71, 1.68)	1.06 (0.69, 1.64)
HOMA-IR ≥4.5	2.09 (1.53, 2.86)	1.52 (1.10, 2.09)
Weight *z* score		
2≤ weight *z* <3	0.97 (0.45, 2.11)	1.02 (0.47, 2.22)
Weight *z* ≥3	2.46 (1.14, 5.27)	2.12 (0.98, 4.59)

^a^Adjusted HR was calculated from a model adjusted for pFIB score components (sex, elevated BP, ethnicity, ALT, HOMA-IR, weight *z* score)

Finally, we stratified the analysis by sex. Given the lower risk of type 2 diabetes in boys, this stratification slightly increased the strength of the association for both sexes separately. The pFIB-c at a cut-off score of 0.4 yielded an adjusted HR of 2.83 (95% CI 1.85, 4.33; *p*=0.002) in boys and 3.56 (95% CI 2.19, 5.78; *p*<0.001) in girls (ESM Table 4).

### Association between pFIB-6 score and adipose tissue insulin resistance

In the Belgian Zeepreventorium cohort (*n*=200), the Adipo-IR index was higher in participants with a pFIB-6 score ≥4 (*p*<0.001) (ESM Fig. 2a). Adipo-IR correlated with multiple components of the score, including ALT, HOMA-IR, weight *z* score (all *p*<0.001) and hypertension (*p*=0.048) (ESM Fig. 2b).

## Discussion

In this nationwide cohort study, we demonstrate that a liver-derived composite risk score, pFIB, identifies a subgroup of children with obesity at substantially increased risk of youth-onset type 2 diabetes. The excess risk was particularly pronounced during adolescence, a critical window of metabolic vulnerability, and attenuated in early adulthood. These findings position pFIB not merely as a fibrosis-oriented tool but also as a marker of a high-risk hepato-metabolic phenotype in paediatric obesity.

To our knowledge, this is the first study to demonstrate that a paediatric fibrosis risk score for MASLD is associated with a clinically meaningful longitudinal outcome. Equally importantly, no established validated clinical prediction models are currently available for estimating incident type 2 diabetes risk in youth with obesity [13]. Existing paediatric models focus primarily on diabetes classification at diagnosis or on the subsequent prediction of metabolic trajectories in diabetes [26]. In this context, our findings highlight that pFIB provides important prognostic information.

The more than twofold increased hazard of type 2 diabetes during adolescence among individuals with elevated pFIB scores extends previous observations from the NASH Clinical Research Network [9], in which greater histological severity was linked to metabolic complications. Our findings suggest that children with a higher estimated fibrosis burden represent a subgroup with particularly adverse metabolic trajectories.

To contextualise the metabolic profile captured by the pFIB scores, in an independent cohort we examined their relationship with the Adipo-IR index, itself a well-established marker of metabolic dysfunction. Demonstrating this association with higher pFIB-6 scores supports the interpretation that pFIB reflects a broader hepato-metabolic disturbance rather than isolated liver injury, echoing earlier observations relating elevated liver stiffness to impaired adipose tissue metabolic function [25].

The pFIB scores combine ALT with metabolic variables such as HOMA-IR, weight *z* score and BP, individually established diabetes risk factors. Multiple components of the pFIB scores were positively associated with the risk of incident type 2 diabetes. Elevated ALT levels showed the strongest association, consistent with the close inter-relationship between hepatic steatosis, insulin resistance and glucose dysregulation [7, 8]. The association with baseline hypertension is consistent with the bidirectional association between hypertension and diabetes [27] and shared pathophysiological mechanisms that include arterial stiffening by insulin resistance [28].

Thus, the pFIB scores do not isolate a purely hepatic effect but instead integrate hepatic and systemic metabolic signals into a composite marker that may capture interactions not reflected by individual components alone. Our findings therefore support the concept that a hepato-metabolic risk signature, rather than fibrosis per se, identifies adolescents at elevated risk of youth-onset type 2 diabetes.

Traditional diagnostic metrics such as AUROC were not applied because the clinical prediction model in this study is prognostic rather than diagnostic, and because our survival data structure involves censoring and non-proportional hazards. Survival-adapted AUROC calculations also require choosing a single prediction time point, which is unsuitable for an age-dependent outcome such as youth-onset type 2 diabetes. We therefore relied on HRs and cumulative incidence as the appropriate measures of association.

The risk of diabetes in relation to pFIB score was time-dependent, as higher scores were not associated with a higher risk of diabetes in young adulthood. This should be interpreted with caution, given the low number of diagnoses in young adults and consequent statistical underpowering. Supposing the observation is valid, one possible explanation is that individuals at high risk of MASLD fibrosis develop type 2 diabetes earlier in life (i.e. during adolescence). In addition, the longer the timeframe between the index test and the outcome, the more changes in predisposing factors (including tested variables) can impact the outcome. Furthermore, the transition to adult care might be linked to a lower awareness and screening rate of asymptomatic type 2 diabetes in these young adults, potentially delaying diagnosis and obfuscating the association between type 2 diabetes and pFIB score.

Of note, incorporating sex in the risk score negatively impacted the association with type 2 diabetes development. Male sex is a well-known risk factor for MASLD, both in adults and in children [17, 29, 30], and therefore boys with obesity are at greater risk of MASLD fibrosis. Sexual dimorphism also affects metabolic regulation, with endogenous oestrogens acting as a protective factor for type 2 diabetes [31]. This contributes to a lower incidence of diabetes in premenopausal women compared with men of the same age, an effect that diminishes after menopause. It is therefore intriguing that in this nationwide Swedish cohort, as well as a US-based cohort [9], the risk of diabetes in adolescents was higher for girls than for boys. As onset of type 2 diabetes is rare before the onset of puberty, the earlier pubertal development in girls might contribute to this effect [32].

Our study has several limitations. First, we used diagnostic codes and prescription of glucose-lowering medication from national registers to infer type 2 diabetes. This could have led to misclassification bias if glucose-lowering medications were repeatedly prescribed for other indications (e.g. polycystic ovary syndrome). Second, the pFIB scores analysed in this study represent a snapshot in time, taken at the start of obesity treatment. It is likely that intercurrent events, such as response to treatment, that modify the risk of type 2 diabetes [8] weakened the association with the pFIB scores. A longitudinal follow-up with sequential determination of these scores would strengthen this analysis, in analogy to studies in adults establishing that increases and decreases in liver stiffness are associated with the risk of MALO [14, 33]. Third, as MASLD is associated with other adverse health outcomes such as MALO and MACE, studying the correlation between these and pFIB is of particular importance. Indeed, we recently employed the BORIS cohort to demonstrate that paediatric obesity is associated with a twofold increased risk of MALO [18]. However, the incidence of MALO and MACE in the current study was low, and pFIB scores were only available in a subset of patients. This precluded meaningful statistical analysis. Future studies could answer this research question by revisiting the dataset in 5–10 years when more outcomes will have occurred, given that it takes a long period for severe hepatological and cardiovascular complications to develop [34].

In conclusion, the liver-derived composite pFIB scores identify adolescents with obesity at markedly increased short-term risk of type 2 diabetes. These findings support the integration of liver-derived biomarkers into early risk stratification frameworks for paediatric obesity management. Our findings suggest that future prospective studies will have the potential to significantly enhance pathophysiological understanding and clinical decision-making if they focus on the following areas: validating the association between paediatric hepatic risk scores and incident metabolic complications; and development of risk indices for the prediction of type 2 diabetes in youth.

## Supplementary Information

Below is the link to the electronic supplementary material.

ESM (PDF 466 KB)

## Data Availability

Patient-level data cannot be shared publicly because of third-party data. Given ethical approval, data may be obtained from the Swedish Childhood Obesity Treatment Register via http://www.e-boris.se/in-english/ and the Swedish National Board of Health and Welfare via registerservice@socialstyrelsen.se.

## Abbreviations

Adipo-IR: Adipose tissue insulin resistance
ALT: Alanine aminotransferase
MALO: Major adverse liver outcomes
MASLD: Metabolic dysfunction-associated steatotic liver disease
pFIB: Paediatric liver fibrosis risk score
ULN: Upper limit of normal
