# Supplementary material for: pFIB scores for paediatric MASLD fibrosis are associated with risk of youth-onset type 2 diabetes in children with obesity
Source: Diabetologia. 2026 Jul 22;69(10):2774–83. doi: 10.1007/s00125-026-06804-4 (PMC13541798; doi:10.1007/s00125-026-06804-4)
Supplement: Supplementary file 1 [file 125_2026_6804_MOESM1_ESM.pdf]

pFIB scores for paediatric MASLD fibrosis are associated with risk of youth-onset type 2 diabetes in children with obesity

Sander Lefere[1,2]*, Resthie R. Putri[3,4]*, Pernilla Danielsson[3], Thomas Casswall[3], Claude Marcus[3], Christian A. Hudert[5], Anja Geerts[1,2], Ruth De Bruyne[6#], Emilia Hagman[3#]

* These authors contributed equally

# Shared senior authors

Affiliations

[1] Hepatology Research Unit, Department of Internal Medicine and Pediatrics, Ghent University, Ghent, Belgium
[2] Liver Research Center Ghent, Ghent University Hospital, Ghent, Belgium
[3] Department of Clinical Science, Intervention and Technology, Karolinska Institutet, Stockholm, Sweden
[4] Department of Medical Epidemiology and Biostatistics, Karolinska Institutet, Stockholm, Sweden
[5] Department of Pediatric Gastroenterology, Nephrology and Metabolic Diseases, Charité - Universitätsmedizin Berlin, Berlin, Germany
[6] Department of Pediatric Gastroenterology, Hepatology and Nutrition, Ghent University, Ghent, Belgium

Contact information

Ruth De Bruyne, MD, PhD
Department of Pediatric Gastroenterology, Hepatology and Nutrition, Ghent University,
Princess Elisabeth Children's Hospital, Corneel Heymanslaan 10, B-9000 Ghent, Belgium
ruth.debruyne@uzgent.be
Tel. +32 9 332 3966

ESM Table 1. Exclusion criteria and the respective ICD-10 codes

Category	Condition	Code
Other causes of liver steatosis	Acute hepatitis A	B15
	Acute hepatitis B	B16
	Other acute viral hepatitis	B17
	Chronic viral hepatitis	B18
	Unspecified viral hepatitis	B19
	Chronic hepatitis	K73
	Drug-induced liver injury	K71
	Alpha-1-antitrypsin deficiency	E88.0
	Autoimmune hepatitis	K75.4
	Disorders of mineral metabolism, including Wilson disease	E83
	Coeliac disease	K90
	Ulcerative colitis	K51
	Crohn disease	K50
	Cystic fibrosis	E84
	Alcoholic liver disease	K70
	Primary biliary cholangitis	K74.3
	Secondary biliary cirrhosis	K74.4
	Unspecified biliary cirrhosis	K74.5
	Muscle dystrophy	G71
	Cardiomyopathies	I42
	Disorders of carbohydrate metabolism	E74
	Disorders of branched-chain amino-acid and fatty-acid metabolism	E71
	Disorders of aromatic amino-acid metabolism	E70
Secondary causes of hypertension	Cushing's syndrome	E24
	Adrenogenital disorders	E25
	Hyperaldosteronism	E26
	Glomerular diseases	N0
	Coarctation of the aorta	Q25.1
	Congenital renal artery stenosis	Q27.1
Other types of diabetes	Type 1 diabetes	E10
	Pre-existing type 1 diabetes mellitus, in pregnancy, childbirth and the puerperium	O24.0
	Maturity onset of diabetes in the young (MODY)	E13
Syndromes related to obesity	Noonan syndrome	Q87.1E
	Prader-Willi syndrome	Q87.1F
	Russel-Silver syndrome	Q87.1G
	Laurence-Moon-Bardet-Biedl syndrome	Q87.8B
	Down syndrome	Q90
	Turner syndrome	Q96
	Klinefelter syndrome	Q98
	Fragile X syndrome	Q99.2

ESM Table 2. Codes to identify type 2 diabetes

Data Source	Diagnosis / medication	ICD-10/ ATC codes
The National Patient Register	Type 2 diabetes	E11
The National Patient Register	Pre-existing type 2 diabetes mellitus, in pregnancy, childbirth and the puerperium	O24.1
The Prescribed Drug Register	biguanides	A10BA
The Prescribed Drug Register	sulfonylureas	A10BB
The Prescribed Drug Register	sulfonamides	A10BC
The Prescribed Drug Register	antidiabetic combinations	A10BD
The Prescribed Drug Register	alpha glucosidase inhibitors	A10BF
The Prescribed Drug Register	thiazolidinediones	A10BG
The Prescribed Drug Register	DPP4 inhibitors	A10BH
The Prescribed Drug Register	GLP-1 analogues	A10BJ
The Prescribed Drug Register	SGLT2 inhibitors	A10BK
The Prescribed Drug Register	Other antidiabetic	A10BX
The Prescribed Drug Register	Insulin	A10A

ICD-10 (the 10th revision of the International Classification of Diseases) codes were used to obtain diagnoses from the National Patient Register, whereas ATC (Anatomical Therapeutic Chemical) codes were used to obtain medications from the Prescribed Drug Register.

ESM Table 3. Association between the pFIB scores and incidence of type 2 diabetes

	**n T2D**	**Unadjusted HR (95% CI)**	**P**	**Adjusted HR[a] (95% CI)**	**P**
**Age 9-25 years**					
pFIB-6 score					
≤1	26/866	Ref	Ref	Ref	Ref
1.5-2	60/1675	1.25 (0.79 – 1.98)	0.346	1.25 (0.79 – 1.98)	0.347
2.5-3	48/1298	1.35 (0.84 – 2.17)	0.223	1.23 (0.76 – 1.99)	0.398
3.5-4	30/645	1.69 (1.00 – 2.85)	0.051	1.54 (0.91 – 2.61)	0.108
4.5-6	37/372	3.86 (2.34 – 6.38)	<0.001	3.28 (1.98 – 5.44)	<0.001
pFIB-c score					
<0.1	51/1478	Ref	Ref	Ref	Ref
0.1 -<0.2	41/1328	0.89 (0.59 – 1.34)	0.572	0.90 (0.60 – 1.36)	0.633
0.2 - <0.3	32/848	1.11 (0.71 – 1.73)	0.638	1.06 (0.68 – 1.64)	0.810
0.3 - <0.4	10/357	0.85 (0.43 – 1.67)	0.638	0.77 (0.39 – 1.52)	0.447
0.4 - <0.5	15/263	1.75 (0.99 – 3.12)	0.056	1.78 (0.90 – 2.86)	0.111
≥0.5	52/528	2.91 (1.98 – 4.29)	<0.001	2.51 (1.69 – 3.72)	<0.001

Hazard ratios (HR) were estimated using Cox regression. [a] Adjusted for age at baseline and parental type 2 diabetes.

ESM Table 4. Association between the pFIB scores and incidence of type 2 diabetes, stratified by sex

	**Unadjusted HR (95% CI)**	**P**	**Adjusted HR[a] (95% CI)**	**P**
**Male**				
pFIB-6 score				
<4	Ref	Ref	Ref	Ref
≥4	3.19 (2.07 – 4.92)	<0.001	2.77 (1.79 – 4.31)	<0.001
pFIB-c score				
≤0.4	Ref	Ref	Ref	Ref
>0.4	3.24 (2.14 – 4.91)	<0.001	2.83 (1.85 – 4.33)	0.002
**Female**				
pFIB-6 score				
<4	Ref	Ref	Ref	Ref
≥4	2.86 (1.76 – 4.65)	<0.001	2.66 (1.63 – 4.33)	<0.001
pFIB-c score				
≤0.4	Ref	Ref	Ref	Ref
>0.4	4.05 (2.52 – 6.48)	<0.001	3.56 (2.19 – 5.78)	<0.001

Hazard ratios (HR) were estimated using Cox regression. [a] Adjusted for age at baseline and parental type 2 diabetes.

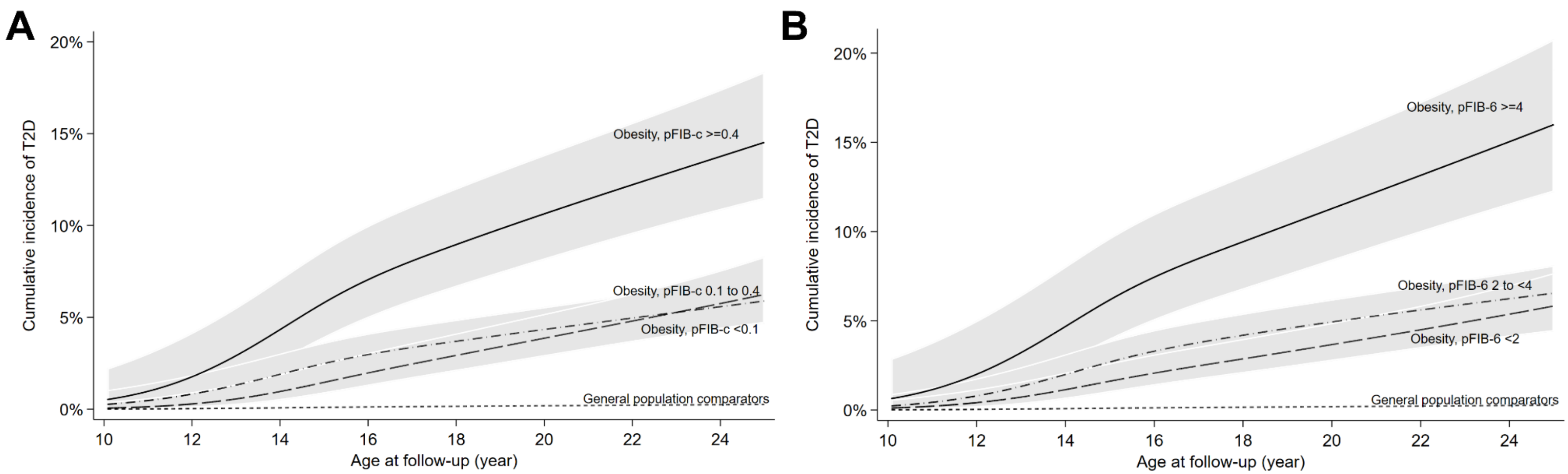

ESM Figure 1. Cumulative incidence of type 2 diabetes by pFIB-c score (A) or pFIB-6 score (B), stratified in low-, intermediate, and high-risk groups as compared to the general population.

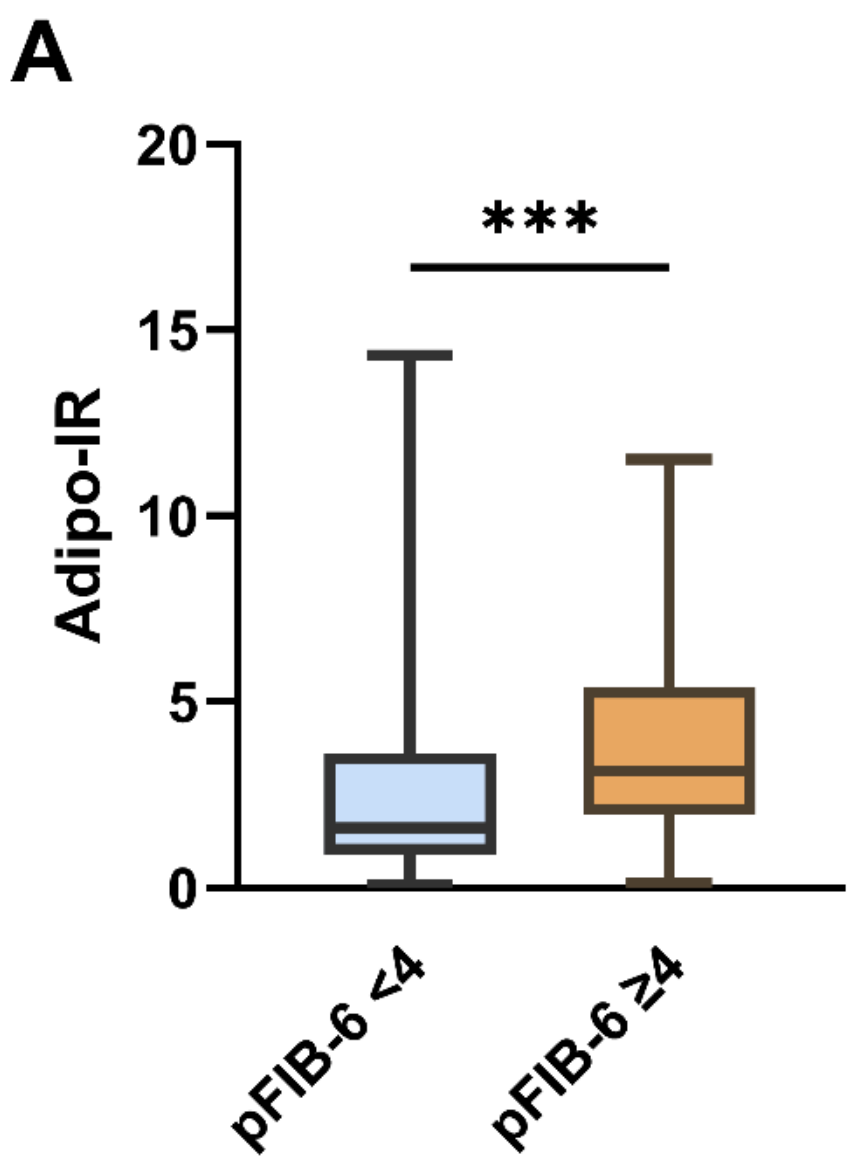

B

Correlation of the pFIB score components with adipo-IR values in the Zeepreventorium cohort (n = 200)

**Variable**	**Spearman coefficient**	**P value**
Sex, male	-0.050	0.478
Hypertension	0.142	0.048
Black ethnicity	0.035	0.633
ALT	0.266	< 0.001
HOMA-IR	0.728	< 0.001
Weight z-score	0.264	< 0.001

ESM Figure 2. The Adipo-IR index according to the pFIB-6 score (A); correlation between the adipo-IR and pFIB score components (B).
